# Adenocarcinoma in Caroli's Disease Treated by Liver Transplantation

**DOI:** 10.1155/1993/61048

**Published:** 1993

**Authors:** J. Balsells, C. Margarit, E. Murio, J. L. Lazaro, R. Charco, M. T. Vidal, J. Bonnin

**Affiliations:** 1 Liver Transplantation Unit Department of Surgery Hospital General Vall d'Hebrón Universidad Autónoma Barcelona Spain; 2 Liver Transplantation Unit Department of Pathology Hospital General Vall d'Hebrón Universidad Autónoma Barcelona Spain; 3 Unidad de Trasplante Hepático Servicio de Cirugía General Hospital General Vall d'Hebrón Paseo Vall d'Hebrón s/n 08035 Barcelona Spain

## Abstract

Caroli's disease is characterized by congenital cystic dilatation of the intrahepatic bile ducts. In 7% of casea a malignant tumor develops complicating the course of the disease.

We report the case of a 25 year-old woman in whom Caroli's disease was diagnosed at the age of 11. From that time on, she had several episodes of cholangitis. In 1989, the abdominal ultrasound and CT scan showed dilatation of the intrahepatic bile ducts, intracystic lithiasis and a solid mass. FNA cytology showed a papillary adenocarcinoma. At laparotomy a tumor was found occupying both hepatic lobes, and intraoperative US showed another two nodules in the left lobe. The tumor was considered unresectable. Examination of the hilar lymph nodes was tumor-negative. Two weeks later, the patient underwent an ortothopic liver transplantation (OLT). The pathological examination confirmed Caroli's disease with adenocarcinoma. Two years after OLT, the patient is alive with normal liver function and no evidence of disease.

To our knowledge this is the first case report of adenocarcinoma in Caroli's disease treated by OLT.


*HPB Surgery*, 1993, Vol. 7, pp. 81-87
Reprints available directly from the publisher
Photocopying permitted by license only

© 1993 Harwood Academic Publishers GmbH
Printed in the United States of America

### CASE REPORT

### ADENOCARCINOMA IN CAROLI'S DISEASE TREATED BY LIVER TRANSPLANTATION

J. BALSELLS, C. MARGARIT, E. MURIO, J.L.LAZARO, R. CHARCO, M.T.VIDAL** and J. BONNIN*

*Liver Transplantation Unit, Department of Surgery and Department of Pathology***

*Hospital General Vall d'Hebrón, Universidad Autónoma, Barcelona, Spain*

*(Received 16 October 1992)*

Caroli's disease is characterized by congenital cystic dilatation of the intrahepatic bile ducts. In 7% of casea a malignant tumor develops complicating the course of the disease.

We report the case of a 25 year-old woman in whom Caroli's disease was diagnosed at the age of 11. From that time on, she had several episodes of cholangitis. In 1989, the abdominal ultrasound and CT scan showed dilatation of the intrahepatic bile ducts, intracystic lithiasis and a solid mass. FNA cytology showed a papillary adenocarcinoma. At laparotomy a tumor was found occupying both hepatic lobes, and intraoperative US showed another two nodules in the left lobe. The tumor was considered unresectable. Examination of the hilar lymph nodes was tumor-negative. Two weeks later, the patient underwent an ortothopic liver transplantation (OLT). The pathological examination confirmed Caroli's disease with adenocarcinoma. Two years after OLT, the patient is alive with normal liver function and no evidence of disease.

To our knowledge this is the first case report of adenocarcinoma in Caroli's disease treated by OLT.

KEY WORDS: Caroli's disease, adenocarcinoma, intrahepatic biliary tumor, liver transplantation

### INTRODUCTION

In 1958, Caroli and Couinaud[3] reported a congenital disease of the liver characterized by cystic dilatation of the intrahepatic bile ducts. Intracystic lithiasis, recurrent cholangitis, and liver abscess are the most common complications associated with Caroli's disease. Less often, in 7% of cases, a malignant tumor develops, complicating the course of the illness[1,4,5,6,15].

We report a case of Caroli's disease with an unresectable adenocarcinoma which was treated with total hepatectomy and liver transplantation. To our knowledge, this is the first case of liver transplantation for malignancy complicating Caroli's disease that has been described.

Address correspondence to: Dr J. Balsells, Unidad de Trasplante Hepático, Servicio de Cirugía General, Hospital General Vall d'Hebrón, Paseo Vall d'Hebrón s/n 08035 Barcelona, Spain

### CASE REPORT

A 25 year-old woman was diagnosed at laparotomy as having Caroli's disease at the age of 11. From that time on, she had several episodes of fever and upper abdominal pain.

In August 1989, she suffered another episode of right upper quadrant pain, radiating to the scapulor associated with fever and chills. An abdominal ultrasound examination followed by a CT scan (Figure 1) showed dilatation of the intrahepatic bile ducts, with intracystic lithiasis. A hyperechogenic solid mass filled some right lobe cysts. Gallbladder lithiasis and a moderately dilated common bile duct were also found. Fine needle aspiration cytology of the mass disclosed a papillary adenocarcinoma.

The biochemistry parameters were: Hb 10.7 g/100 ml, WBC 12.900, PT 76%, BUN 26 mg/100 ml, glucose 83 mg/100 ml, total bilirubin 0.4 mg/100 ml, AST 9 IU/l, ALT 12IU/l, AP 252 IU/l, total proteins 7.4 g/100 ml, albumin 3.9 g/100 ml, and cholesterol 154 mg/100 ml. Blood cultures were negative.

A laparotomy performed to evaluate the feasibility of liver resection and to rule out the possibility that the tumor had spread outside the liver. Mild dilatation of the common bile duct, cholelithiasis, some lymphadenopathy at the porta hepatis, and a mass in the right lobe, infiltrating the left hepatic lobe, were found. An intraoperative ultrasonographic examination confirmed the presence of a tumor in the right hepatic lobe, infiltrating segment IV. Two hyperechogenic nodules were also discovered in segment III compatible with malignancy. Conventional hepatic resection was not possible. There was no evidence extrahepatic spread of the tumor and lymph node pathology was negative. The tumor was considered unresectable and a liver transplantation was proposed; this was accepted by the patient.

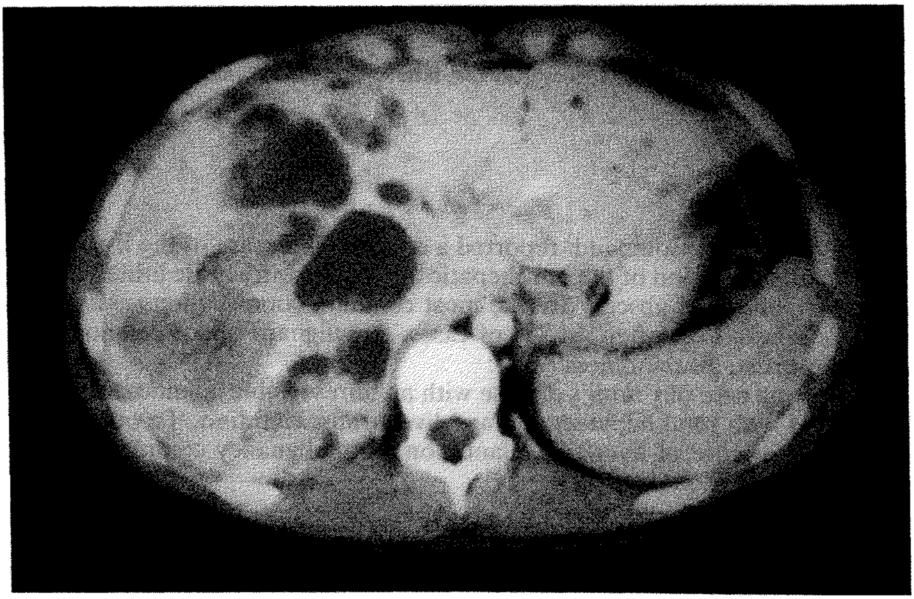

**Figure 1** CT scan: Bile duct dilatation, stones and a mass filling some right lobe cysts.

Eighteen days after the exploratory laparotomy, total hepatectomy and liver replacement was performed. No veno-venous by-pass was needed, and biliary reconstruction was with a Roux-en-Y choledochojejunostomy.

Pathological examination of the liver confirmed cystic dilatation with lithiasis, cholangitis and adenocarcinoma of the intrahepatic bile ducts (Figures 2 and 3), reactive inflammation in the hilar lymph nodes was found.

Immunosuppression induction consisted of low-dose cyclosporine, prednisone and prophylactic OKT3 for 5 days. At present the patient is being treated with cyclosporine and prednisone.

The patient's postoperative course was uneventful; a mile rejection episode responded to steroids. The patient was discharged 26 days after liver transplantation with normal hepatic function. She is now alive and well, and shows no evidence of tumor recurrence 2 years after transplantation.

### DISCUSSION

Various authors provide evidence to support the concept of Caroli's disease as a premalignant condition[6,8]. Fozard[8] reported a case complicated by severe displasia without malignancy; this situation may be considered the first step in the degeneration of Caroli's disease. Cholangiocarcinoma has been reported in approximately 7% of patients with this disease[1,6], although Phinney[12] found a 14% incidence of malignancy.

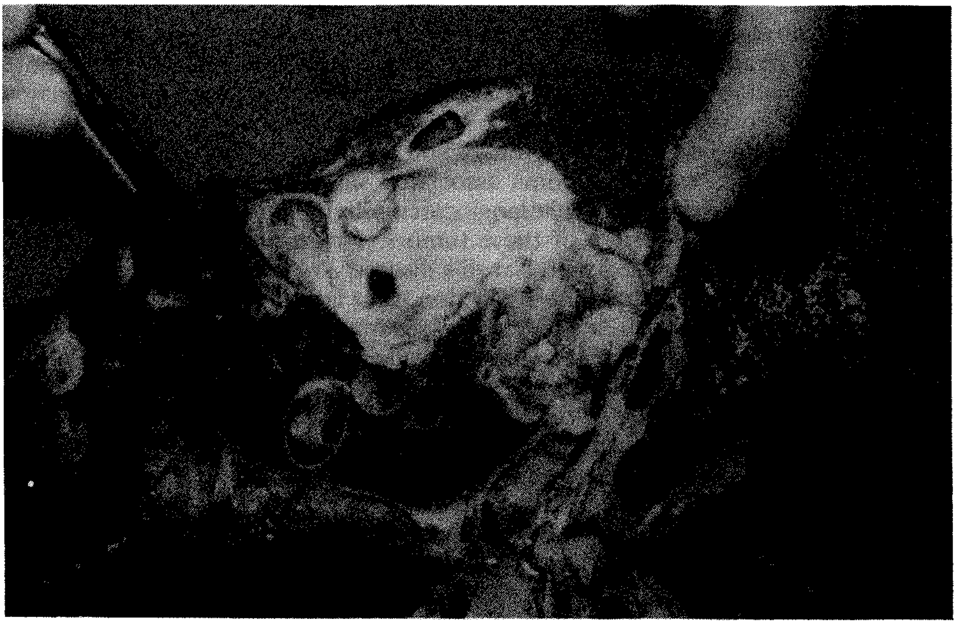

**Figure 2** Liver specimen showing biliary cysts with lithiasis and tumor.

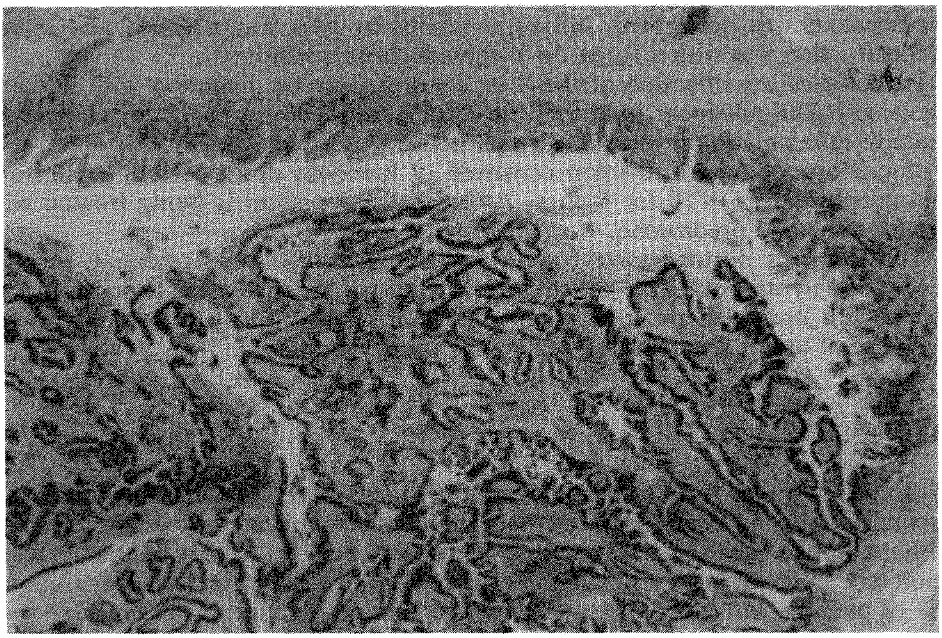

**Figure 3** Microphotography: papillary adenocarcinoma of bile ducts.

Most of the cases of adenocarcinoma in Caroli's disease were diagnosed at necropsy[6,14] or as an incidental finding in the pathologic examination of a specimen from liver resection[4,15]. At present, Caroli's disease is diagnosed with imaging techniques, but possible complications[14], such as degeneration, may be diagnosed earlier with fine needle aspiration cytology and liver biopsy guided by ultrasound.

The majority of cases of carcinoma associated with Caroli's disease have been described as adenocarcinoma or cholangiocarcinoma. The bile duct epithelium is clearly implicated in the origin of these tumors. However, hepatocellular carcinoma has also been associated with Caroli's disease[2].

From a therapeutic standpoint, a distinction must be drawn between the limited and total forms of Caroli's disease. The localized forms, even those associated with complications, are curable by surgery, and hepatic resection should be aggressively performed in these selected patients[5,9,10,15].

Forms involving both lobes of the liver pose difficult therapeutic problems. The standard techniques in cases without carcinoma involve T-tube drainage of the common bile duct or internal drainage by biliodigestive anastomosis[9]. However, these procedures often result in incomplete decompression of the biliary tree since the diseased intrahepatic ducts frequently have stenotic segments that cause bile stasis, recurrent infection, and intrahepatic stones. Another problem is that congenital cellular aberrations that resulted in the original cystic malformation may have a predisposition to undergo neoplastic changes independent of bile stasis. Supporting this idea is the observation that malignant degeneration often occurs in cystic areas despite apparently adequate drainage by patent surgical biliary-enteric anastomosis[6,7]. The interval between the drainage procedure and the subsequent development of carcinoma is usually less than 10 years, and in Flanigan's review averaged 4 years[6,7]. There appears to be a 50% risk of malignant disease in patients who have undergone a previous biliodigestive drainage procedure[7].

It has been stated that the treatment for Caroli's disease with carcinoma is liver resection, when feasible. Usually, the involvement of the tumor in both lobes, or the widespread alteration of the liver parenchyma produced by the disease, precludes partial hepatectomy. In these cases liver transplantation is the only possibility for treatment, when extrahepatic tumor spread has been ruled out by a pretransplant laparotomy with pathologic examination of the hilar lymph nodes[13].

Commonly, liver transplantation for Caroli's disease is indicated in the diffuse form of the illness with recurrent cholangitis, and when secondary biliary cirrhosis complicates the disease. Bearing in mind the relatively high incidence of malignant degeneration after a bilio-digestive drainage procedure[7], liver replacement seems the best therapeutic option for diffuse, advanced Caroli's disease.

In early liver transplantation trials, a primary hepatic malignancy that could not be removed by the conventional technique of subtotal hepatic resection was thought to be an unequivocal indication for liver replacement. Enthusiasm for this approach has been dampened in several major centers by the high recurrence rates (ranging from 50 to 80%) of the original malignancies. Long-term survival rates of approximately 20-30% have been reported from different centers[11,13].

An important prognostic factor for the proper selection of patients, in order to reduce the incidence of tumor recurrence after transplantation, is the stage of the tumor[13]. The rate of survival in lymph-node positive stages is far lower than in lymph-node negative stages. The 6-month, 1-year and 2-year actuarial survival data in the Hannover experience[13] for lymph node-negative hepatocellular carcinoma patients were 83%, 75% and 75%, whereas in the lymph node-positive cases, results were 33%, 11% and 11%. In lymph node-negative bile duct carcinomas results were 100%, 100% and 83%, whereas when lymph node-positive, they were 40%, 13% and 0%.

Thus, according to present knowledge, one may consider the lymph node-negative tumor stage, with no other signs of extrahepatic tumor growth, to be an indication for liver grafting. A pretransplant laparotomy would clarify the diagnosis and investigate lymph node involvement, providing information for the resectability decision. This pretransplant exploratory laparotomy may be performed at the transplant laparotomy with another receptor ready in the case of lymph node involvement.

It is not known if adenocarcinoma associated with Caroli's disease has a long-term prognosis different from other bile duct carcinomas because, to our knowledge, this is the first report of liver grafting for a neoplasic complication of this disease.

### *References*

1. Bloustein, P.A. (1977) Association of carcinoma with congenital cystic conditions of the liver and bile ducts. *Am.J.Gastroenterol.*, **67**, 40–46
2. Caroli, J. and Corcos, V. (1964) La dilatation congénitale des voies biliaires intra-hépatiques. *Rev.Med.Chir.Mal.Foie*, **39**, 1–7

3. Caroli, J., Couinaud, D., Saupault, R., Porcher, P. and Etéve, J. (1958) Une affection nouvelle sans doute congénitale des voies biliaires. La dilatation unilobaire des canaux hépatiques. *Sem.Hop.Paris*, **34**, 136–143
4. Chen, K.T.K. (1981) Adenocarcinoma of the liver. Association with Congenital Hepatic Fibrosis and Caroli's disease. *Arch.Pathol.Lab.Med.*, **105**, 294–295
5. Chevillotte, G., Sastre, B., Sahel, J., Payan, H., Michotey, G. and Sarles, H. (1984) Maladie de Caroli localiseé et associeé à un adénocarcinome pappillaire mucosécrétant. Intérêt de la résection hépatique. *Presse Med.*, **13**, 1137–1139
6. Dayton, M.T., Longmire, W.P. and Tompkins, R.K. (1983) Caroli's Disease: A premalignant condition? *Am.J.Surg.*,**145**, 41–48
7. Flanigan, D.P. (1977) Biliary carcinoma associated with biliary cysts. *Cancer*, **40**, 880–883
8. Fozard, J.B.J., Wyatt, J.I. and Hall, R.I. (1989) Epithelial dysplasia in Caroli's disease. *Gut*, **30**, 1150–1153
9. Mercadier, M., Chigot, J.P., Clot, J.P., Langlois, P. and Lansiaux, P. (1984) Caroli's Disease. *World J.Surg.*, **8**, 22–29
10. Nagasue, N. (1984) Successful treatment of Caroli's disease by hepatic resection. *Ann.Surg.*, **200**, 718–723
11. O'Grady, J.G., Polson, R.J., Rolles, K., Calne, R.R. and Williams, R. (1988) Liver transplantation for malignant disease. *Ann.Surg.*, **207**, 373–379
12. Phinney, P.R., Austin, G.E. and Kadell, B.M. (1981) Cholangiocarcinoma arising in Caroli's disease. *Arch.Pathol.Lab.Med.*, **105**, 194–197
13. Ringe, B., Wittekind, C., Bechstein, W.O., Bunzendahl, H. and Pichlmayr, R. (1989) The role of liver transplantation in hepatobiliary malignancy: A retrospective analysis of 95 patients with particular regard to tumor stage and recurrence. *Ann.Surg.*, **209**, 88–98
14. Rogstad, K., Freeman, J., Moorghen, M. and Record, C.O. (1986) Difficulty in diagnosing complications of Caroli's disease. *J.Clin.Gastroenterol.*, **8**, 582–585
15. Roudot-Thoraval, F., Zafrani, E.S., Hannoun, S., Fagniez, P.L., Métreau, J.M. and Dhumeaux, D. (1983) Résection hépatique pour maladie de Caroli limiteéau foie gauche. Découverte fortuite d'un cholangio-carcinome. *Gastroenterol.Clin.Biol.*, **7**, 319–320

*(Accepted by S. Bengmark 21 January 1993)*

### INVITED COMMENTARY

Balsells *et al.* describe the first case of successful liver transplantation for nonresectable cholangiocellular carcinoma arising from Caroli's disease in a 25 year old patient. Due to chronic inflammation cystic dilation of the intrahepatic bile ducts is known to predispose to development of adeno- or less frequently hepatocellular and squamous cell carcinoma. From the literature the authors report an incidence of 7%. Generally, prognosis of cholangiocellular carcinoma is very unfavourable. Our own experience with liver transplantation for nonresectable cholangiocellular carcinoma in 14 patients showed a median survival rate of 4 months. All patients surviving more than 30 days died from tumor recurrence. This is worse than in other hepato-biliary malignancies and has to be differentiated sharply from proximal bile duct carcinoma of the hilar type with a much better prognosis — in our series 4 patients survived more than 5 years. Therefore, with special regard to the limited availability of donor organs, the indication for liver transplantation, even in the case of a lymph node negative stage, has to be discussed critically and we should perform an extended resection whenever feasible.

We agree with the authors that resection with partial hepatectomy is the treatment of choice especially for localized symptomatic Caroli disease without tumor. Even in bilateral disease partial hepatectomy can be discussed because in some of our patients considerable relief from cholangitis was observed. The typical indication for liver transplantation should be related to secondary biliary cirrhosis and impairment of liver function. So far, we have performed liver transplantation in 3 patients with advanced biliary cirrhosis in one case combined with renal transplantation for chronic glomerulonephritis. Two patients are alive 1.5 and 4 years after transplanttion. With regard to the risk of carcinoma in Caroli's disease and similarly in primary sclerosing cholangitis, there is a dilemma about the decision to transplant as malignancy usually cannot be diagnosed in an early stage. Carcinoma has to be suspected in case of sudden clinical deterioration and increasing jaundice, accompanied by elevation of tumormarkers CEA or CA 19/9 in the serum. However, clear criteria for the optimum time for transplantation cannot be defined yet. One of our patients who had undergone partial hepatectomy for Caroli's disease subsequently developed carcinoma. Due to rapid tumor progress the patient died before liver transplantation could be performed.

The excellent result of Balsells *et al.* presenting a patient who is alive for more than two years after transplantation without evidence of tumor recurrence has led the authors to suggest that Caroli associated cholangiocellular carcinoma might have a better prognosis. This hypothesis has to be elucidated in the future.

Arved Weimann and Burckhardt Ringe

